# American pika in a low-elevation lava landscape: expanding the known distribution of a temperature-sensitive species

**DOI:** 10.1002/ece3.1626

**Published:** 2015-08-13

**Authors:** Matt Shinderman

**Affiliations:** Department of Forest Ecosystems and Society, Oregon State University Cascades CampusBend, Oregon

**Keywords:** American pika, climate adaptations, Newberry National Volcanic Monument, occupancy, *Ochotona princeps*, thermal maxima

## Abstract

In 2010, the American pika (*Ochotona princeps fenisex*) was denied federal protection based on limited evidence of persistence in low-elevation environments. Studies in nonalpine areas have been limited to relatively few environments, and it is unclear whether patterns observed elsewhere (e.g., Bodie, CA) represent other nonalpine habitats. This study was designed to establish pika presence in a new location, determine distribution within the surveyed area, and evaluate influences of elevation, vegetation, lava complexity, and distance to habitat edge on pika site occupancy. In 2011 and 2012, we conducted surveys for American pika on four distinct subalpine lava flows of Newberry National Volcanic Monument, Oregon, USA. Field surveys were conducted at predetermined locations within lava flows via silent observation and active searching for pika sign. Site habitat characteristics were included as predictors of occupancy in multinomial regression models. Above and belowground temperatures were recorded at a subsample of pika detection sites. Pika were detected in 26% (2011) and 19% (2012) of survey plots. Seventy-four pika were detected outside survey plot boundaries. Lava complexity was the strongest predictor of pika occurrence, where pika were up to seven times more likely to occur in the most complicated lava formations. Pika were two times more likely to occur with increasing elevation, although they were found at all elevations in the study area. This study expands the known distribution of the species and provides additional evidence for persistence in nonalpine habitats. Results partially support the predictive occupancy model developed for pika at Craters of the Moon National Monument, another lava environment. Characteristics of the lava environment clearly influence pika site occupancy, but habitat variables reported as important in other studies were inconclusive here. Further work is needed to gain a better understanding of the species’ current distribution and ability to persist under future climate conditions.

## Introduction

Understanding species adaptation to climate change is of paramount importance for conservation and management of biodiversity worldwide. Temperature-sensitive species are of particular concern given observed and anticipated changes in global surface temperatures. In some ecosystems, species have adapted to high daytime temperatures by taking refuge in belowground habitats where temperatures are significantly lower than at the surface (Wolf et al. 1996; Williams et al. 1999; Kearney et al. 2009; Walde et al. 2009; Lagarde et al. 2012; Pike and Mitchell 2013). The extent to which these habitats will remain refugia in a warmer climate is currently unknown.

In the western United States, the American pika (*Ochotona princeps*), a small lagomorph, is regarded as an indicator of species response to climate change in alpine systems (Krajick 2004; Simpson 2009). Pika are thermally sensitive, with a high body temperature and relatively low upper lethal temperature (MacArthur and Wang 1973; Smith 1974). In captivity, temperatures as low as 25.5°C have proved fatal (Smith 1974). Thermo-regulating behaviors among pika include reduced midday activity and the use of cool within-rock microclimates (Smith 1974). The latter seems to be a particularly important characteristic of pika habitat selection, wherein cool microclimates created by talus and talus-like environments provide insulation, runways, and areas for food caching (Smith 1974; Smith and Weston 1990).

American pika are distributed throughout the mountainous regions of western North America (Smith and Weston 1990; Galbreath et al. 2009; Millar and Westfall 2010). In some portions of their range, pika have experienced significant range retraction following the Last Glacial Maximum, with their habitat becoming increasingly restricted to sky islands (Galbreath et al. 2009). In the Cascades, it is thought that pika expanded northward into Canada as the continental ice sheet receded (Galbreath et al. 2009). The species is often described as being confined to alpine environments, typically no lower than 2500 m in the southern part of its range (Smith and Weston 1990). More recent work indicates that pika are more widely distributed at lower elevations, including in lava flows, road cuts, and rock quarries (Beever et al. 2008; Simpson 2009; Millar and Westfall 2010; Rodhouse et al. 2010; Manning and Hagar 2011; Collins and Bauman 2012; Millar et al. 2013).

Recent discoveries of pika in nonalpine environments, where temperatures regularly exceed thermal maxima for the species, challenge assumptions about the species’ habitat preferences, distribution, and adaptability. Similar to other thermally sensitive species, pika apparently persist in these environments using belowground microhabitats that buffer high surface temperatures. Relatively little is known about these habitats, particularly their long-term viability as thermal refugia under future climate scenarios. Determining the viability of low-elevation pika populations requires a better understanding of the species’ current distribution, relative abundance, and microhabitat preferences.

Pika habitat occupancy is influenced by several factors, including temperature, precipitation, vegetation cover, and elevation (Beever et al. 2003, 2010; Millar and Westfall 2010; Rodhouse et al. 2010; Wilkening et al. 2011). The relative influence of these habitat features appears to vary by location (Jeffress et al. 2013). Rodhouse et al. (2010) developed a predictive occupancy model to evaluate the influence of habitat characteristics (elevation, lava complexity, and vegetation cover) on pika occurrence at Craters of the Moon National Monument (CRMO). Elevation and lava complexity were the strongest predictors, although forb cover was also important. No pika were detected below 1600 m, and they were more likely to be found in structurally complex pahoehoe lava than in other lava types (Rodhouse et al. 2010).

By contrast, Millar and Westfall (2010) did not find evidence of a relationship between elevation and patch occupancy. At predominantly nonlava sites in the Oregon Cascades, Sierra Nevada, and central and southwest Great Basin, pika appeared to tolerate a wider range of temperatures and precipitation than commonly believed. The authors suggested that little is known about the subsurface matrix environments pika inhabit. Similarly, more recent work suggests that near-surface temperatures, growing season precipitation, and subsurface water sources are strongly correlated with pika occurrence (Erb et al. 2011; Beever et al. 2013).

Pika tend to forage within close range of den and nest sites, especially at higher temperatures (Smith 1974). In areas with relatively high forb cover, pika have shown a preference for forb species, particularly during the growing season (Huntly et al. 1986; Dearing 1997). Likelihood of pika occupancy has been shown to increase with greater forb cover (Rodhouse et al. 2010; Wilkening et al. 2011). In addition to preferences for vegetation types, pika foraging tends to decline with distance from talus cover (Huntly et al. 1986; Roach et al. 2001). The effect of distance to edge of talus or talus-like cover on pika site occupancy has not been evaluated in nonalpine environments.

American pika do not hibernate in winter, and in some habitats individuals store food in haypiles to meet nutritional requirements when plant material is not readily available (Conner 1983; Smith and Weston 1990). The type of vegetation stored in haypiles varies by location and is influenced by site characteristics, including summer high temperatures, moisture availability, and quality of available forage (Wilkening et al. 2011; Smith and Erb 2013). In low-elevation environments where forage is available year-round, pika may not create haypiles (Simpson 2009; Varner and Dearing 2014a).

This study examined the occupancy patterns and habitat characteristics of a recently discovered pika population on the lava flows of Newberry National Volcanic Monument (NNVM) in Central Oregon. These flows occur at an elevation well below the 2500 m lower limit for pika distribution reported in the previous literature. We tested models of pika occupancy using vegetation cover, lava complexity, and elevation as predictor variables. Additionally, the distance from sample plots to lava edge was included as a predictor in our models. Model results were used to evaluate the following hypotheses:

H_1_: The odds of encountering pika increase with increasing lava complexity. Surface openings and micro-topographic variation trap moisture, increase shading and provide cool microclimates favored by pika. As lava complexity increases these features become more abundant;

H_2_: The odds of encountering pika increase with increasing forb cover relative to total vegetation cover. Forbs provide high nutritional value during the growing season when pika are actively foraging, and forb cover has been an important predictor of pika occupancy in other studies;

H_3_: Pika occupancy will be positively influenced by elevation, where the odds of encountering pika increase with increasing elevation. In other low-elevation lava environments (e.g., Craters of the Moon) elevation was an important predictor of pika occurrence, presumably due to changes in temperature and available moisture;

H_4_: The odds of encountering pika increase as the distance between survey locations and the edge of lava flows decreases. Total vegetation cover tends to decline with increasing distance from the edge of the lava flows surveyed. Lava complexity also tends to be high near the lava edge, creating favorable habitat conditions for pika.

**Figure 1:**
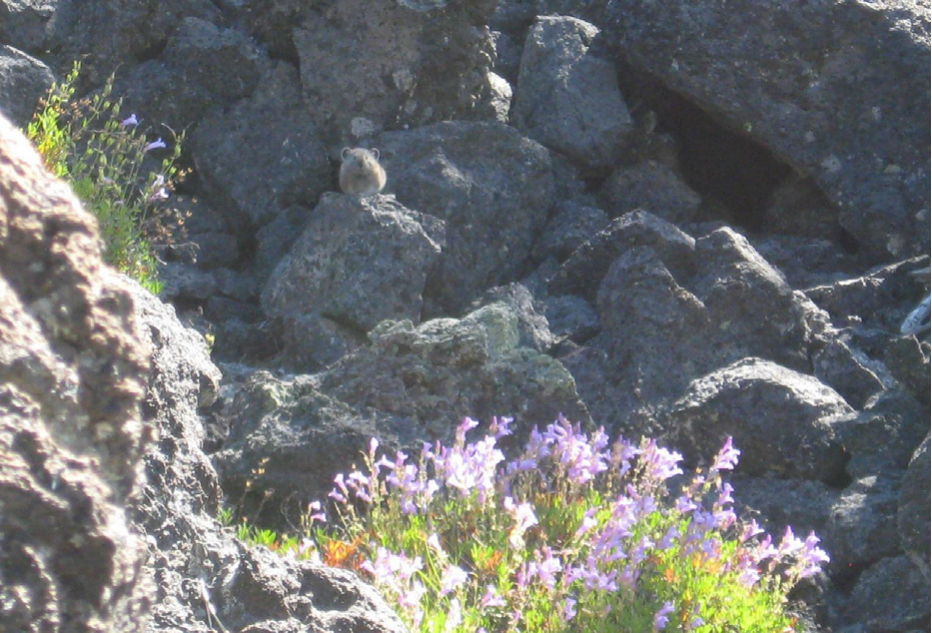
American pika at the Lava Butte flow, Newberry National Volcanic Monument, Oregon.

The primary objectives of this study were to: (1) document pika occurrence in a newly reported, nonalpine environment; (2) evaluate the degree to which occupancy models developed in other lava environments accurately explain habitat occupancy for this population; and (3) determine the relative influence of habitat variables on pika site occupancy within the Monument (Fig.1).

## Materials and Methods

### Study area

**Figure 2:**
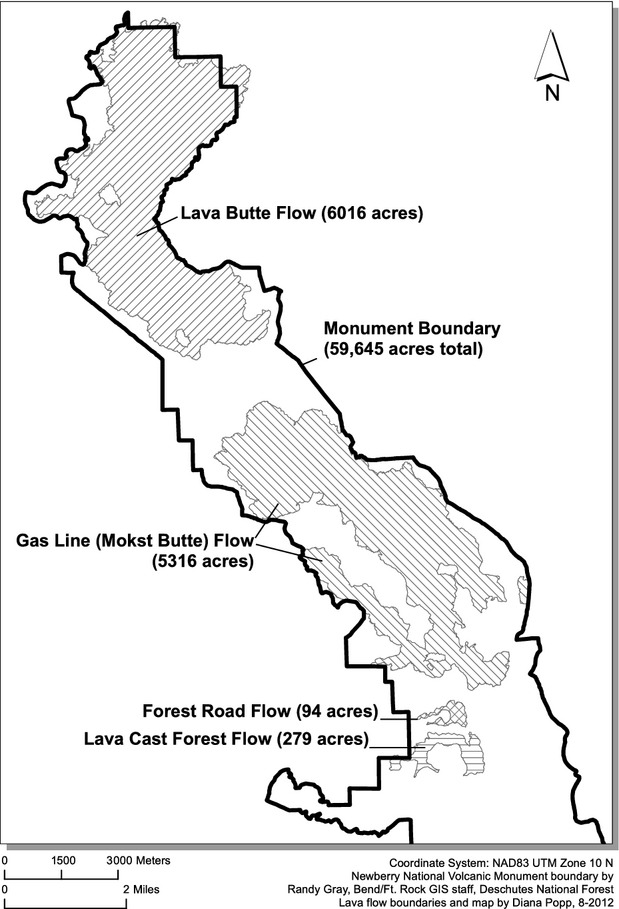
Lava flows included as sampling areas for survey of American pika in Newberry National Volcanic Monument, Oregon.

**Figure 3:**
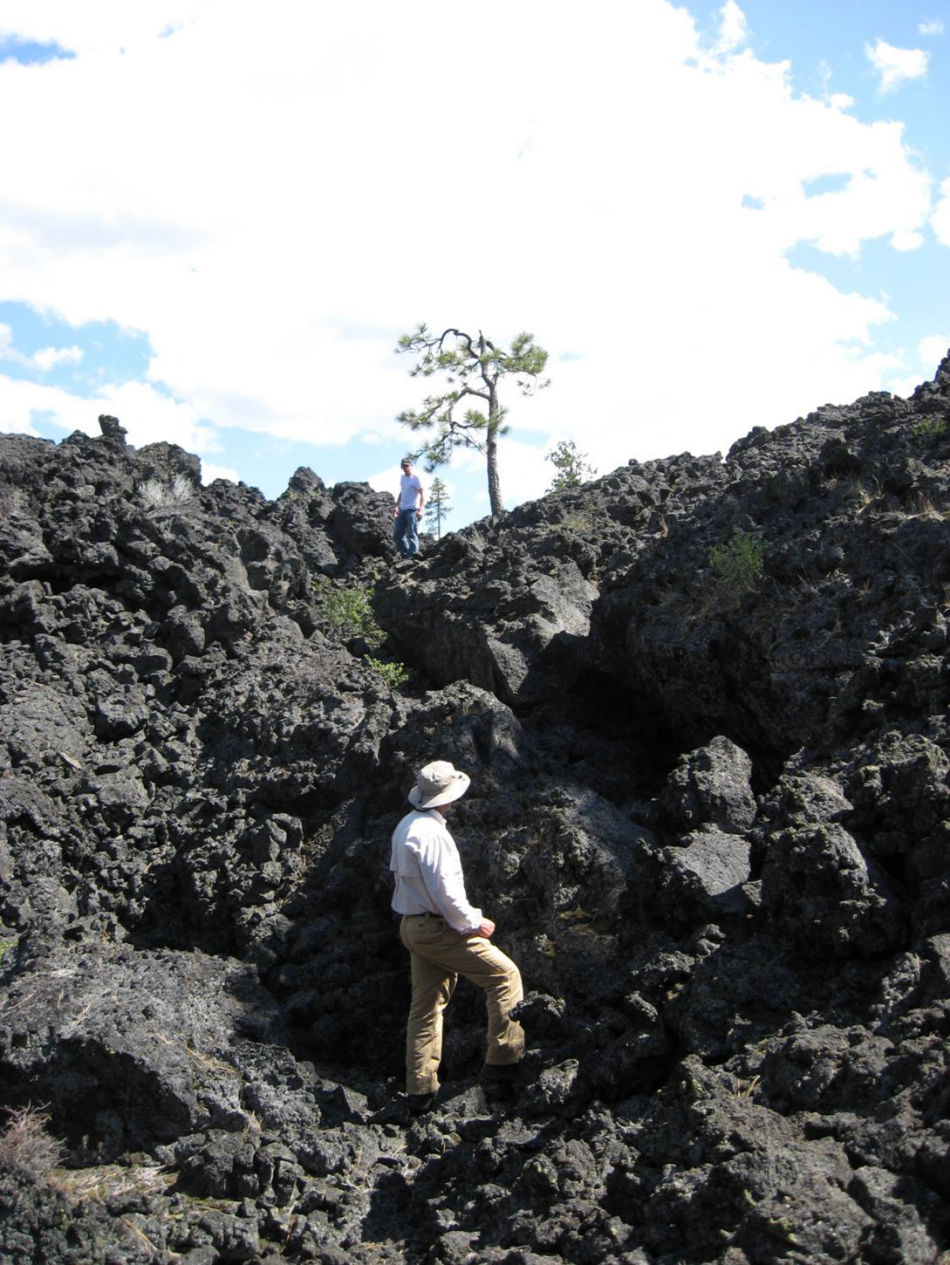
Complex lava environment at the Lava Butte flow at Newberry National Volcanic Monument, Oregon.

Our study was conducted at 146 sites in the NNVM (43°41′39″N, 121°15′7″W), which encompasses 225 km^2^ within the Deschutes National Forest in Central Oregon. The Monument is comprised of the Newberry Caldera itself as well as most of the volcano's Northwest Rift Zone, a 30-km-long system of fissures and vents extending northwest from the caldera, including the Lava Butte cinder cone (Donnelly-Nolan et al. 2011). We focused on four basalt and basaltic andesite flows, ranging from approximately 6000 to 7000 years in age, located along the rift zone (Fig.2). Sites ranged in elevation from 1210 to 1783 m. Lava flows within the Monument consist primarily of broken ‘a’ā lava of varying complexity, with patches of pāhoehoe-type lava, extensive large fissures and lava tree casts (Fig.3).

Newberry National Volcanic Monument falls within the northern portion of the Mazama Ecological Province. Precipitation patterns vary substantially by location within this portion of the Province, primarily in relation to elevation and the Cascades rain shadow (Anderson et al. 1998). Climate data for the Monument are collected at Lava Butte (1417 m), the source of the lowest elevation lava flow surveyed for this study. Average annual precipitation for Lava Butte was 455 mm for the period 2002–2012. For the same period, average winter maximum and minimum temperatures were −18.8 and 20.5°C, respectively, while average growing season maximum and minimum temperatures were −3.3 and 38.1°C. Regular climate monitoring does not occur for other areas on the Monument. Data collected in the 1990s indicate that annual precipitation on the caldera floor (1935 m) can be nearly double precipitation at the lowest elevations within Monument boundaries (Morgan et al. 1997).

Vegetation cover varies between and within flows. The interior of the Lava Butte Flow is largely barren, with the exception of widely scattered patches of dense shrub cover (primarily *Arctostaphylos patula*) in areas where soils are more developed. Vegetation cover and species diversity tend to increase along an elevational gradient, wherein the higher elevation flows (with higher annual precipitation) support more vegetation cover, greater plant species diversity, and more established plant communities.

Vegetation on all four flows consists of a variety of shrubs, with wax currant (*Ribes cereum*) and rock spirea (*Holodiscus dumosus*) dominant. Rabbitbrush (*Ericameria nauseosa*) is also common. Forb cover is limited in most areas of the lava flow, with hot rock penstemon (*Penstemon deustus*) and Davidson's penstemon (*Penstemon davidsonii*) dominant. Round-leaf alumroot (*Heuchera cylindrica*) is also common above 1490 m, especially at the lava edge. Small patches of grasses, typically Idaho fescue (*Festuca idahoensis*), can be found on all flows and particularly on north-facing slopes. The soil of the region is principally composed of deposits of eolian pumice and other volcanic material over basaltic bedrock (Anderson et al. 1998).

### Sampling design

Lava flows on the Monument are characterized by extremely variable and complicated terrain. Access to the flows is limited to unimproved forest roads maintained by the Deschutes National Forest. In some areas, particularly the Lava Butte flow, the study area is bordered by private property and the Deschutes River, further limiting access. With the exception of one short trail at the Lava Lands Visitor Center and a utility road bisecting one of the flows, the interior of the four lava flows sampled is only accessible by foot over very rough terrain. Travel safety issues and limited accessibility made a census approach impractical.

Pika presence was determined using a probabilistic sampling design in four distinct lava flows within Monument boundaries, comprising 4738 ha of lava habitat. We developed spatially balanced sample locations for each flow using the RRQRR software extension in ARC GIS version 9.3 (Theobald et al. 2007). Sample locations were defined by an average territory size of 452 m^2^, consistent with the most recent pika sampling methodology used in lava environments (Rodhouse et al. 2010). Minimum distance between survey sites was set to 24 m to ensure independent samples, resulting in a nearest-neighbor distance of 40 m between survey points generated by the GIS. The four sampling areas encompassed 2435, 2152, 38, and 113 ha, respectively.

We followed the survey methodology utilized by Rodhouse et al. (2010) to allow for comparison of results to the study at CRMO. Surveys for pika presence entailed location of sample points using a GPS and then establishing a 12-m radius from the center of the plot, marking boundaries with rock cairns. Each site was searched by a single observer for approximately 30 min (compared to 20 min at CRMO) within the marked boundary, with time split between silent observation for pika visual or audible detection and active searching for pika sign (feces and haypiles). Feces were recorded as either old or fresh based on appearance and texture (Nichols 2010). Haypile material was recorded as current year or old based on green material content.

Pika presence was characterized as one of three categories: no sign, old sign, and detected (new sign combined with visible or aural detection). In contrast to Rodhouse et al. (2010), we did not revisit all survey locations to estimate detection probability. Pika detection probability was assumed to be similar to other studies in similar lava environments (>90%; Ray and Beever 2007; Rodhouse et al. 2010; Beever et al. 2010); however, a small subsample of sites (*n* = 20) was visited twice by separate observers to ensure consistency of observations. Percent vegetation cover within the 12 m survey area was established via ocular estimation (Daubenmire 1959) for graminoids, forbs, shrubs, and total cover. Lava complexity at each site was qualitatively characterized as either low, moderate, or high. Low lava complexity sites contained little vertical relief (<1 m), no large surface openings, and small lava fragments. Moderate complexity sites exhibited greater vertical relief (1–2.5 m), some surface openings and lava fragments up to 0.5 m^2^. High complexity sites featured substantial vertical relief (>2.5 m), numerous large surface openings, and larger lava fragments (>0.5 m^2^). Distance to edge of lava flow was estimated in ArcGIS by overlaying an ortho-rectified image of each flow over the sample point image.

Prior to field studies, all field researchers were trained to ensure consistency of data collection, in particular emphasizing vegetation cover estimation, evaluation of pika scat age and the basic survey approach. In June 2011, we conducted a pretest of the sampling methodology on the Lava Butte flow based on an initial draw of 96 samples from ArcGIS data. No pika or pika sign was detected in sample locations beyond 400 m of the lava edge. To ensure safety and reasonable access, the available sampling area for each flow was redefined to within 400 m. of the lava edge and areas easily accessible by road. Modification of survey boundaries resulted in a total of 146 sample locations between the four flows. The total area surveyed in the four flows encompassed 1001 (Lava Butte), 748 (Mokst Butte), 38 (Forest Road), and 36 (Lava Cast Forest) ha, respectively.

Surveying occurred from June to August 2011 and during the same time period in 2012. Pika tend to exhibit a crepuscular activity pattern, so data collection was restricted to morning hours (7 am to noon) to coincide with peak pika activity. For 2011, all 146 sites were sampled, and data from 145 sites were used for analysis (one site was excluded due to incomplete information). We reduced the total number of sample sites to 124 in 2012 based on access issues experienced the previous year.

In addition to pika occupancy surveys, we deployed Maxim iButton temperature sensors at 22 locations (18 at Lava and Mokst Buttes and four at Forest Road Flow) where pika were detected in 2011 and 2012. Sensors were inserted into 12-inch-long sections of PVC pipe predrilled for air circulation and capped at both ends. At each sensor deployment location, we positioned one sensor unit at the lava surface and another 1 m below the surface in a crevice/opening in the immediate vicinity of pika sign. Belowground units were lowered into rock crevices with parachute cord secured at the surface by a rock or stick anchor. Sensors were set to record continuous temperature data every 60 min for 4 days. Temperature data collection occurred from July to September 2011 and 2012.

### Analysis

We tested four models to evaluate the influence of habitat characteristics on pika occurrence based on models used in previous work in lava environments (Rodhouse et al. 2010). Specifically, we evaluated relationships between elevation, lava complexity, vegetation cover, and distance to lava edge as factors that may influence site occupancy at a given location. Data from all four sampling locations were pooled for each year. Elevation and distance to edge were mean-centered and standardized (e.g., Z-scores) for easier interpretation and improved model fit. We used the information criterion function in multinomial logistic regression (IBM Corp. 2013) to compute Akaike information criterion (AIC). AIC_c_ was then calculated using the formula AIC_c_ = AIC + {2*k*(*k *+* *1)/*n-k*-1} where *k* is the number of model parameters and *n* is sample size (Burnham and Anderson 2002). We selected AIC_c_ for model comparison because of the low number of pika reported in each of the four sampling areas. We computed Nagelkerke's generalized R^2^ to provide an estimate for the proportion of variance explained by model parameters (Nagelkerke 1991). Differences between above and belowground temperatures were evaluated using an independent samples *t*-test.

## Results

### Pika occupancy

Pika were detected at 37 sites in 2011, representing 26% of sampling locations. An additional 27 incidental sightings or aural detections (outside plot boundaries) were recorded during site visits. Although the rate of pika detection increased from lower to higher elevation sites (from 16% to 38%), pika and pika sign were present at all four lava flows. The total number of pika detected was nearly identical between the four flows, with nine pika per flow detected at sample locations on the Lava Butte, Mokst Butte, and Forest Road flows, and ten detected at the Lava Cast Forest flow. The majority (73%) of pika detections occurred in the most complicated lava formations, and specifically sites which contained numerous surface openings and substantial vertical complexity compared to surrounding areas. Without exception, all new pika sign, sightings, and vocalizations occurred within 200 m of the lava edge (Figs.7). Of those, 38% were detected within 50 m. Pika detected in the interior of flows were exclusively limited to sites within or in the immediate vicinity of kipukas, areas of land with established plant communities surrounded by lava.

**Figure 4:**
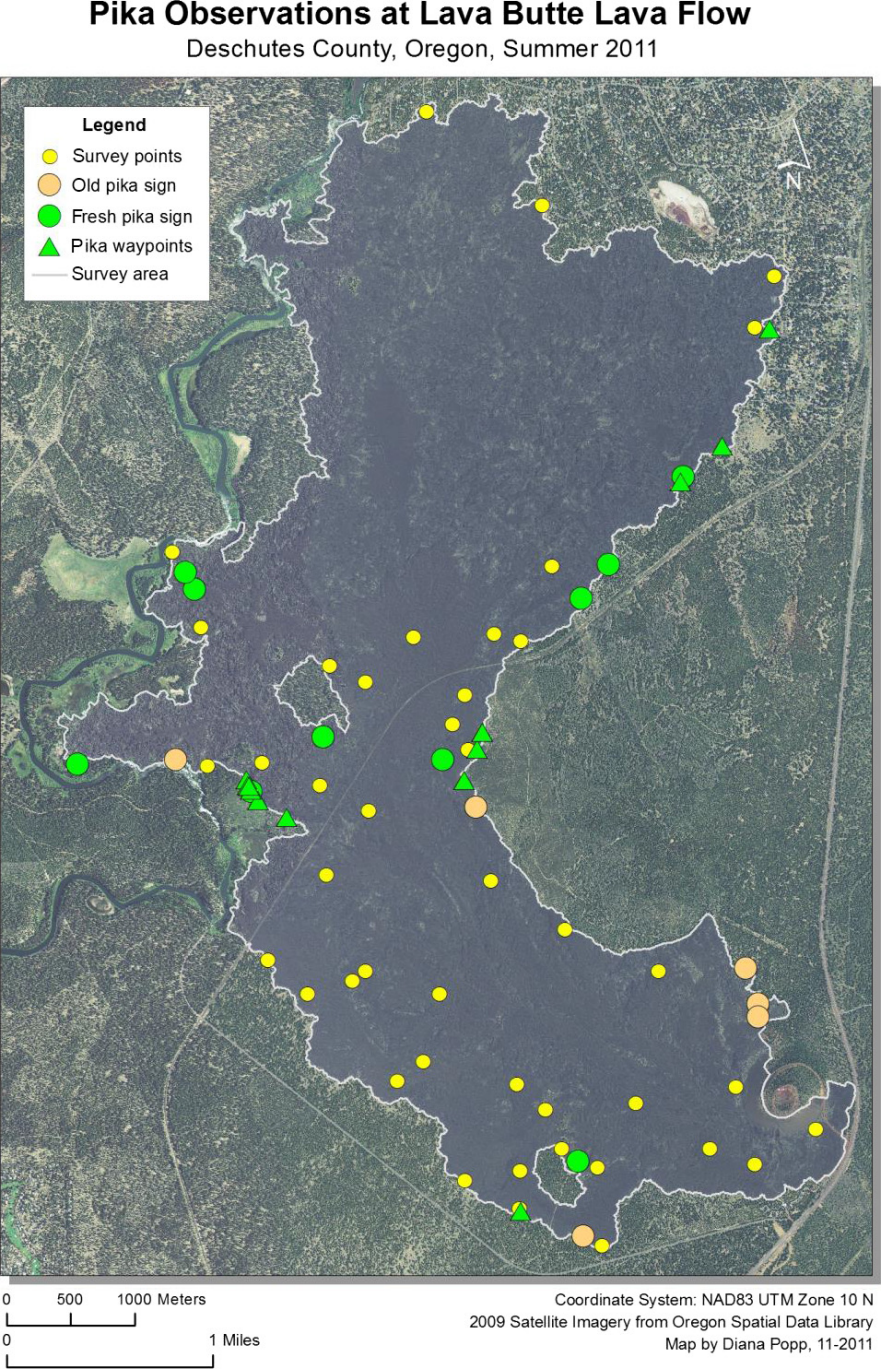
American pika survey locations and detections at Lava Butte flow within Newberry National Volcanic Monument, Oregon, 2011.

**Figure 5:**
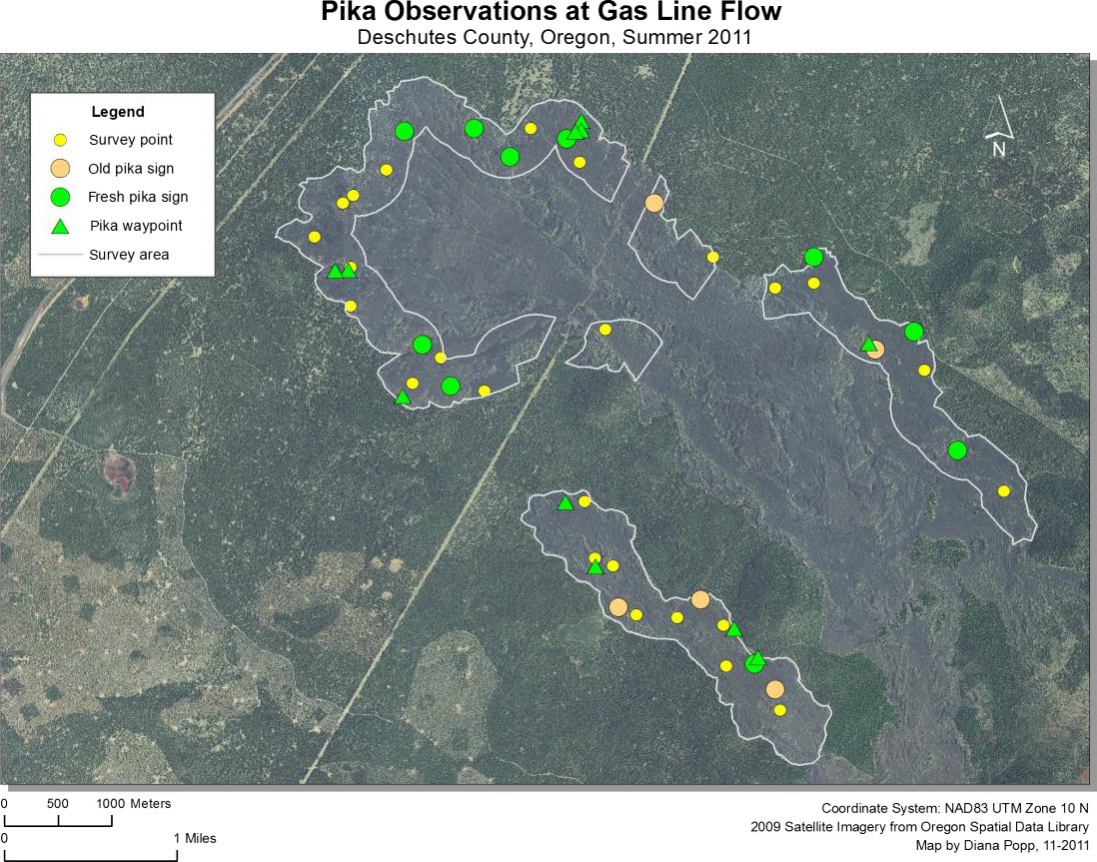
American pika survey locations and detections at Mokst Butte flow within Newberry National Volcanic Monument, Oregon, 2011.

**Figure 6:**
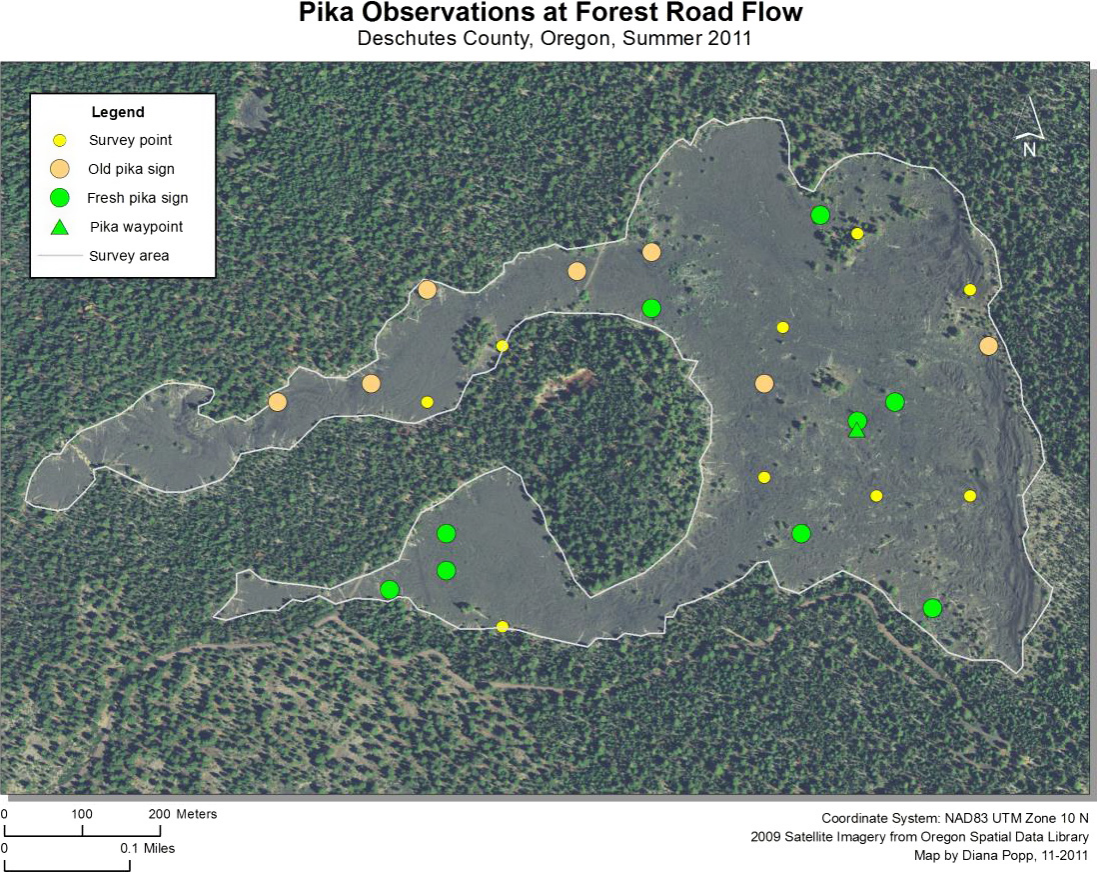
American pika survey locations and detections at Forest Road flow within Newberry National Volcanic Monument, Oregon, 2011.

**Figure 7:**
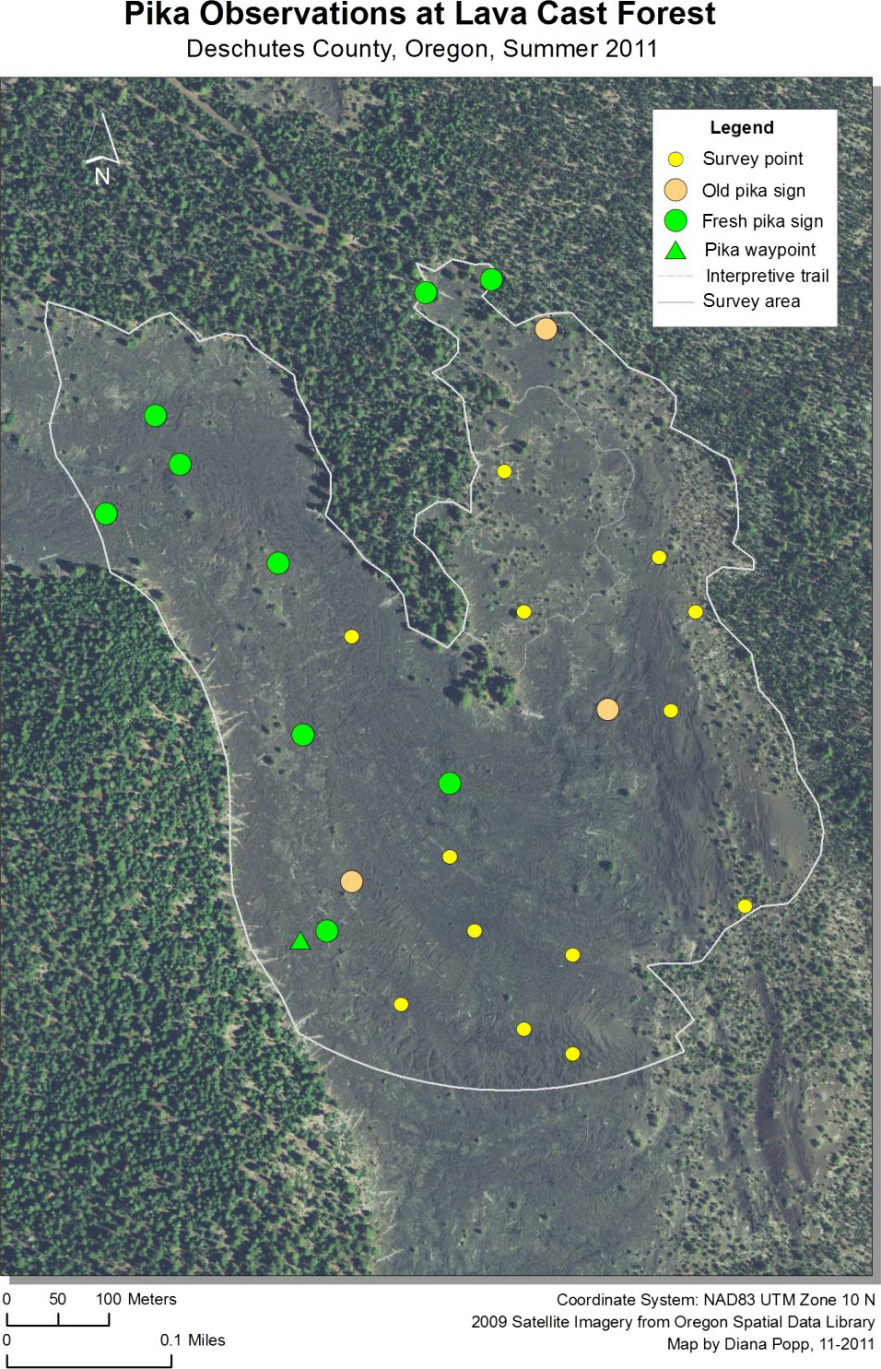
American pika survey locations and detections at Lava Cast Forest flow within Newberry National Volcanic Monument, Oregon, 2011.

In 2012, pika were detected at 27 sample sites (19%) and at 47 locations outside plot boundaries. Similar to the previous year, pika detection increased with elevation (14–28%) and the majority (89%) of detections occurred in the most complicated lava formations. Several pika were detected beyond 200 m from the lava edge, but the majority (56%) were located within 100 m.

### Detection site temperature data

**Figure 8:**
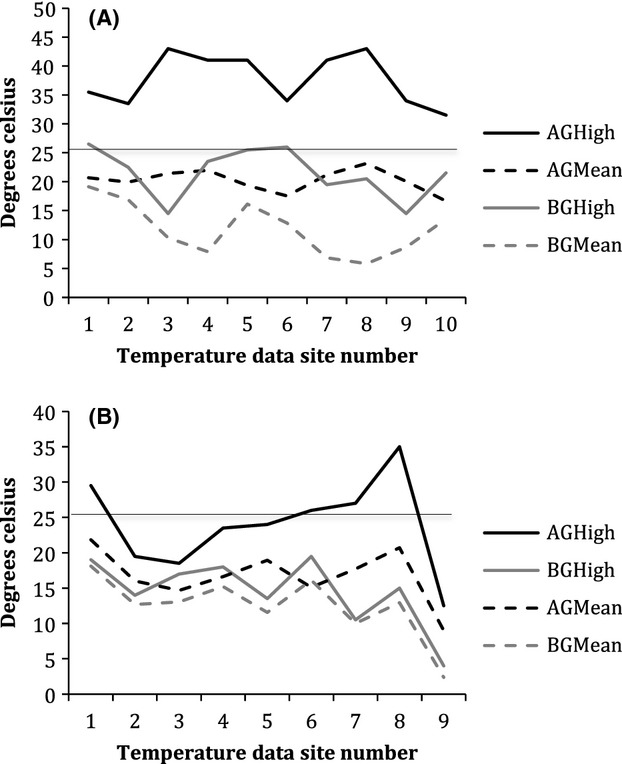
Above (AG) and belowground (BG) high temperatures at pika detection sites at Newberry National Volcanic Monument, Oregon. 2011 data (A) were collected from sites at the Lava Butte flow. Data for 2012 (B) were collected from sites at Lava Butte (sites 1-6) and Mokst Butte (sites 7-9). The solid line at 25.5°C indicates the predicted thermal maxima for pika.

Three sensors were incorrectly retrieved after 24 h (rather than 48) but continued to collect temperature data after removal from the field. As a result, they were omitted from the analysis, leaving 19 recordings. Above and belowground temperatures varied substantially across sensing locations (Fig.8A and B). In 2011, aboveground temperatures (M = 37.5°C, SD = 4.44) were significantly different (*t* = −8.31, *P* = 0.000) from belowground temperatures (M = 21.5°C, SD = 4.34). The maximum day-time surface temperature recorded was 43°C while the belowground temperature at the same site was 21°C. The mean difference between above and belowground high temperatures across all sites was 16.3°C. Belowground temperatures remained at or below the thermal maxima reported for pika at all sample locations. A similar trend was observed in 2012, where high temperatures aboveground (M = 23.9°C, SD = 6.60) were significantly higher (*t* = 3.45, *P* = 0.003) than high temperatures (M = 14.5, SD = 4.90) below the surface. Mean difference between above and belowground high temperatures across sites was 9.4°C.

### Model testing

Model performance varied between the two sampling years but generally offered relatively low predictive value in terms of pika occurrence. Chi-square statistics for both years were significant, indicating that all models accounted for more variability in pika occurrence than chance alone. None of the models produced an *R*^*2*^ equivalent higher than 0.33 (Table1). For 2011, the model which best fit the data included elevation, lava complexity, and distance to edge (Table1). All three variables had a statistically significant (*P* < 0.05) influence on the likelihood of pika occurrence, although elevation and lava complexity had the most substantial impacts (Table2).

**Table 1:** Model fit performance for pika occurrence at Newberry National Volcanic Monument in 2011 and 2012. Models are ranked using Akaike information criterion (AIC_c_) and Akaike weights (*w*_*i*_). k is the number of parameters in the model, and *R*^2^_*N*_ is Nagelkerke's generalized *R*^2^. Parameters for each model are predictor variables for elevation (mean-centered and standardized), lava complexity, vegetation cover, and distance to edge of lava (mean-centered and standardized)

Model variables	ΔAICc	*w*_*i*_	*k*	*R* ^2^_*N*_
2011				
Elevation + Lava complexity + Distance to edge	0.0	0.57	4	0.22
Elevation + Lava complexity + Cover + Distance to edge	1.14	0.32	5	0.24
Elevation + Lava complexity	3.88	0.08	3	0.13
Elevation + Lava complexity + Cover	7.00	0.03	4	0.18
2012				
Elevation + Lava complexity + Cover	0.0	0.68	4	0.32
Elevation + Lava complexity	2.10	0.24	3	0.26
Elevation + Lava complexity + Cover + Distance to edge	4.41	0.07	5	0.33
Elevation + Lava complexity + Distance to edge	8.19	0.01	4	0.27

**Table 2:** Predictor variable weights on odds of American pika occurrence for the best performing models explaining pika occupancy at Newberry National Volcanic Monument. Variables are considered significant at *P* < 0.05. Exp(B) coefficients represent the odds ratio for detecting pika relative to changes in predictor variable values

Predictor variable	SE	Sig.	Exp(B)
2011			
Elevation	0.295	0.007	2.22
Lava complexity	0.375	0.017	2.45
Distance to edge	0.361	0.035	0.468
2012			
Elevation	0.345	0.005	2.63
Lava complexity	0.503	0.000	7.03
Cover	0.026	0.008	1.07

Pika were 2.2 times more likely to occupy sites at higher elevations (Table2). Similarly, the odds of encountering pika were 2.5 times higher in the most complicated lava types compared to areas with low vertical complexity and fewer surface openings. The probability of encountering pika in the most complicated lava types was 44%. By comparison, the probability associated with pika occurrence at a given elevation was quite low (7% for the elevation with the greatest standard deviation from the mean).

**Figure 9:**
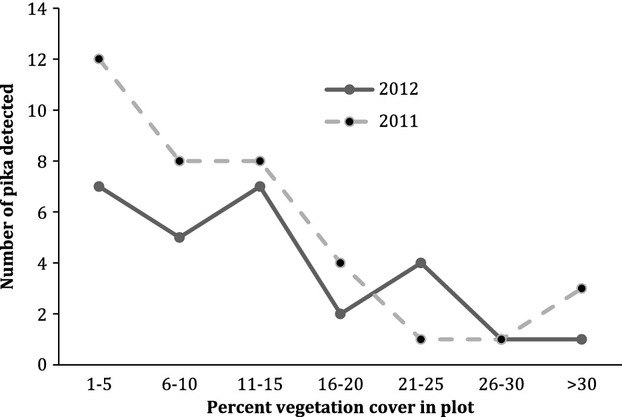
Number of American pika detected in relation to percent vegetation cover at survey sites within Newberry National Volcanic Monument, Oregon, 2012.

Lava complexity and elevation remained important as predictors of pika occurrence in 2012, but the best fitting model included vegetation cover and omitted distance to edge (Table1). The odds of pika occurrence with elevation change were similar to results from 2011, but the influence of lava complexity was greater. Pika were seven times more likely to occur in areas of high lava complexity compared to less complex sites. The probability of pika occurrence at sites with high lava complexity was 40%. The influence of vegetation cover, while significant, was less clear. Increasing vegetation cover was not associated with increasing frequency of pika occurrence (Fig.9). In fact, 70% of pika detections in 2012 occurred at sites with less than 15% total vegetation cover.

## Discussion

This study documents persistence of a newly discovered population of American pika at elevations below those predicted as optimal for the species. Like other lava environments where pika have been recently documented, lava flows at NNVM appear to be serving as thermal refugia for pika, despite summer temperatures which regularly exceed thermal maxima for the species. It is likely that pika inhabit other low-elevation lava flows in areas that have never been surveyed.

The predictive occupancy model developed by Rodhouse et al. (2010) for pika at CRMO worked reasonably well for patterns of occupancy observed at NNVM. In fact, the two most important factors influencing site occupancy, lava complexity, and elevation were the same for the two locations. Although there were similarities in terms of model parameter performance, overall model performance (as a function of *R*^*2*^*N* values) was lower at NNVM. Additionally, some variables which were significant for models applied at CRMO had equivocal impacts in our models.

On the four lava flows we surveyed, sites of higher lava complexity were significantly more likely to be occupied. As a nonburrowing species, American pika utilize broken rock features and crevices (Smith 1974; Smith and Weston 1990), so more surface openings provide more available habitat. Although this study supports the notion that lava complexity has a strong influence on pika site occupancy, we cannot attribute pika occurrence to specific aspects of complexity. Pika may respond to a number of habitat cues related to substrate, including topographic variation/shading, number of surface openings per unit area, depth of crevices, and connectivity of crevices belowground.

Complex lava environments, and in particular those with greater relief, may experience reduced solar exposure and create more moderate microclimates for pika. Jeffress et al. (2013) determined that topographic position (and associated solar exposure) was positively associated with pika site occurrence across a range of sites in the western United States. In that study sites located on steeper, north-facing slopes were more likely to be occupied than other sites. We did not record site topographic position for this study, although we did find pika in sites oriented toward all four cardinal directions.

Complex lava formations may also be influenced by cool air masses stored below the surface. Lava tubes, ice caves, and other subsurface voids are notable features of the lava flows at NNVM and throughout Central Oregon. These features are often characterized by consistent cool air which can be expelled from surface openings with pressure. It is possible that fractures within the more complicated lava formations connect surface openings to substantial reservoirs of cool air deeper within lava flows.

Similar to studies in other lava environments (e.g., Varner and Dearing 2014b), we observed substantial cool air flow from surface crevices at a number of sites where pika were detected. Although rock-ice features and subsurface water have been described as important factors associated with pika occurrence in nonlava habitats (Millar and Westfall 2010; Erb et al. 2011, 2013; Wilkening et al. 2015), it is not currently known whether ice or water is present underneath the flows where pika were detected at NNVM. In any case, surface feature connectivity to subsurface voids may be an important component of microhabitat suitability for pika in these environments.

The relationship between elevation and pika site occupancy is difficult to interpret, partially because elevation is a proxy for environmental variables which directly influence species habitat preferences. At CRMO, no pika were found at sites below 1600 m, but the occupancy study conducted there provides little definitive information to explain why. In contrast, Jeffress et al. (2013) documented fresh pika sign at 1259 m at Lava Beds National Monument (LABE), which is similar to NNVM in terms of elevation ranges (∼1200–1700 m) and macroclimate. Pika were detected at the lowest elevations (∼1200 m) on the Lava Butte flow, in the immediate vicinity of the flow's terminus at the Deschutes River. Under current climate conditions, elevation does not seem to be a limiting factor for pika at NNVM.

As mentioned above, elevation is often used as a proxy for more specific variables that influence occurrence of species across the landscape, including microhabitat temperatures (Korner 2007). Similar to other studies (Beever et al. 2008, 2010; Millar and Westfall 2010; Wilkening et al. 2011; Varner and Dearing 2014b), we found substantial variation in above and belowground temperatures at all locations where temperature data were collected. More importantly, temperatures belowground approached the suggested thermal maxima for pika in only two of 19 locations. It is clear that microhabitat variables within the lava flows surveyed currently mitigate the effects of surface temperatures on pika occurrence at NNVM.

Vegetation cover, particularly forb cover, has been reported as an important predictor of pika occurrence in other areas (Rodhouse et al. 2010; Wilkening et al. 2011; Jeffress et al. 2013). We found little clear evidence for a strong relationship between vegetation cover and pika site occupancy at NNVM. Vegetation cover at sites where pika or pika sign were detected ranged from zero to 60%, with the majority (76%) characterized by less than 15% total vegetation cover. Additionally, shrub cover represented the most significant cover type (by percentage) in the majority of pika detection sites. In contrast to previous studies where pika preferentially selected forbs for haypiles (Huntly et al. 1986; Dearing 1997), we observed pika actively foraging on rock spirea, and it was the most common species found in haypiles. Shrub cover was not a significant driver of pika site occupancy in our models, but it may influence pika site selection at NNVM.

The weak relationship between vegetation and pika occurrence may result from several factors. First, it is possible that measurement error associated with ocular estimation by multiple observers influenced modeling results for vegetation cover. Second, establishing a direct linkage between pika habitat requirements and vegetation cover relies on accurate assumptions about territory size. For this study, we adopted the territory size used for pika at CRMO (452 m^2^), another lava environment. Vegetation outside plot boundaries was not included in cover estimates. This sampling approach ignores the distinction between territory size and home range, the latter of which can be particularly important for foraging behavior. Pika territory size has been reported as roughly 55% of home range size (Kawamichi 1982; Ivins 1984; Smith and Ivins 1986). As such, it may be more appropriate to estimate vegetation cover within larger plots defined by home range rather than smaller plots based on territory size.

Access to vegetation within lava flows can be limited, particularly toward the interior. We hypothesized that pika may select sites based on proximity to the lava edge where they can access vegetation off the flow. Our modeling results did not support this hypothesis, even though the majority of pika we detected were within 100 m of the lava edge. By restricting our sampling area to a 400-m buffer from the lava edge we may have applied too fine a filter to detect a distance to edge relationship. Distance to edge may indeed be an important factor influencing pika site selection on lava flows, particularly larger flows. Further work on spatial relationships between pika site selection and distance to edge is warranted.

In some environments, particularly in complex lava, pika detection probability may not be as high as previously reported. For this study, we assumed detection probability would be similar to other studies in lava environments, an approach adopted most recently by Jeffress et al. (2013). We observed substantial variation in pika behavior and habitat usage among the four flows. Pika at the Lava Butte flow vocalized less frequently than their higher elevation counterparts and were generally less tolerant of human activity, often retreating belowground immediately after detection. Additionally, we found little evidence of haypiling at Lava Butte, even at locations where pika sign was readily visible. In contrast, haypiles were detected on the other flows. Follow-up visits indicate that at least some pika at Lava Butte forage aboveground during the winter, reducing the need for haypiling as a winter foraging strategy. Variation in haypiling behavior has been reported in other studies (Beever et al. 2008; Simpson 2009), so these observations are not surprising. In sum, both direct and indirect signs of pika presence may be less reliable in some locations, complicated lava environments in particular.

Combined with other recent work (Simpson 2009; Rodhouse et al. 2010; Manning and Hagar 2011; Jeffress et al. 2013; Varner and Dearing 2014a,b), this study suggests that pika may be more adaptable to a wider variety of habitat conditions than previously reported. Further, variations in behavior observed in our study and elsewhere (Simpson 2009; Varner and Dearing 2014b) may indicate a wider degree of behavioral plasticity than has been credited to the species. There is some degree of convergence in the literature pertaining to habitat characteristics which influence pika site occupancy in general, but drivers of site selection in lava environments remain unclear. The influence of vegetation on pika site selection is particularly nebulous, at least for this location. Additional work on the spatial dimensions of vegetation cover in pika habitat is warranted.

Pika persistence at NNVM stands in contrast to other locations where growing season precipitation and snowpack have been reported as important drivers of occupancy and relative abundance (Erb et al. 2011; Beever et al. 2013). At the lowest elevation sites within NNVM 30-year average growing season precipitation is very low (8 mm), snow rarely persists for longer than a few weeks, and the growing season is short (late June to early September). While the exact mechanisms are not currently known, it is clear that other habitat variables at NNVM compensate for climate conditions which limit pika persistence elsewhere.

Recent discoveries of pika in areas considered to be outside the norm for the species suggest that the norm should be re-evaluated. Although pika have been documented in a handful of nonalpine environments, many potential environments with similar habitat characteristics have yet to be surveyed. It is entirely possible that pika distribution is substantially broader than currently reported, which challenges previous assumptions about the species’ dispersal following the last glacial maximum.
